# Commissioning of the Varian universal interstitial cylinder system for HDR brachytherapy of gynecological cancer

**DOI:** 10.1002/acm2.14605

**Published:** 2024-12-17

**Authors:** Sheridan Meltsner, Oana Craciunescu, Julie Raffi, Casey Lee, Yang Sheng, Junzo Chino, Diandra Ayala‐Peacock, Yongbok Kim

**Affiliations:** ^1^ Department of Radiation Oncology Duke University Durham North Carolina USA

**Keywords:** applicator commissioning, GYN HDR brachytherapy, universal interstitial cylinder

## Abstract

**Purpose:**

This paper outlines the commissioning of the Varian (VMS, Varian Medical Systems, Palo Alto, CA) Universal Interstitial Cylinder (UIC) applicator set for Ir‐192 HDR brachytherapy. The UIC was commissioned for use with CT and MRI and a custom phantom was designed to avoid the introduction of water‐like materials into the needle guide tracks. Various marker strands were investigated to determine which allowed the most accurate reconstruction of source positions.

**Methods:**

Planar kV and MV imaging, along with physical measurements and autoradiographs, were used to commission the physical dimensions of all components of the UIC applicator set. CT and MR imaging was used to further commission one configuration of the UIC with UCP and eight interstitial needles in a simulated clinical setup using a GYN phantom. Three different methods of channel identification were compared – no radio opaque markers, VMS numbered markers, or nylon coated stainless steel leader wires – to see which best aided in channel identification and image registration. An HDR MRI Lumen marker (C4 Imaging, LLC) was used to verify any applicator rotation on MR scans during image registration. Three types of GYN phantoms were investigated – wet towel, gelatin, and ground beef. Dimensions of all components were compared with vendor provided information, including the solid applicator models, which are based on the computer‐aided design model files of the specific applicators.

**Results:**

The dimensions of the applicators could be validated using physical measurements, kV and MV planar imaging, and CT scans. The ground beef based GYN phantom best eliminated the introduction of water into the needle guide tracks that was found when using a water or gel‐based phantom. CT scans using no radio opaque markers did not allow the plastic needles to be visualized well enough to digitize source positions. CT scans with VMS markers showed significant artifact. CT scans with the nylon coated stainless steel wires provided the best visibility of the needle locations to aid in digitizing source positions. The use of an MR marker allowed the channel to be identified on the MR scan and confirm rotation for image registration.

**Conclusions:**

The UIC set and applicator configuration was commissioned for CT and MR based treatment planning. The plastic components of the UIC applicator set pose challenges to the commissioning process but the use of radio opaque markers seen on CT combined with MR image registration allow the source positions within the needles, as well as the location of the end of the needles, to be digitized appropriately. A ground beef phantom minimized the fluid introduced into the needle guide track, minimizing any unintended MR and CT signal in the needle guide tracks.

## 1 INTRODUCTION

The Varian Universal Interstitial Cylinder (UIC) system available from Varian Medical Systems, Inc. (VMS, Palo Alto, CA) is designed for Ir‐192 HDR brachytherapy to provide a hybrid (intracavitary cylinder + interstitial needles ± tandem) solution for the treatment of gynecological cancer sites such as vagina, vaginal stump, cervix, uterus, and endometrium. The UIC system consists of a set of UIC vaginal cylinders with available diameters of 25, 30, 35, and 40 mm (Table 1). The UIC is designed to be used with up to eight (25 and 30 mm UIC) or ten (35 and 40 mm UIC) plastic interstitial needles with either sharp tips or blunt tips (Table 1). Each interstitial needle is 2 mm in diameter and 320 mm in length. In addition to the needles, the center channel of the UIC is used with any one from a set of either plastic Universal Cervix Probes (UCP; Table 1) or Universal Titanium Cervix Probes, or with a rigid guide tube (previously commissioned) if the use of a cervix probe is not indicated. As the titanium probes are not MR safe and MR is routinely used as part of the treatment planning process in our clinic, we only purchased and commissioned the UCP probes, the UIC, and the plastic interstitial needles which are all MR conditional. The collets, located on the proximal end of the UIC and used to secure the needles at the proper depth in tissue, and the mandarins, used to stiffen the needle during insertion, are also not MR safe. The mandarins are not present during the MR scan and the collets are securely attached to the device and located outside the MR scan range. In physicians’ preference, we acquired and commissioned the sharp tip rather than the blunt tip plastic interstitial needles and acquired and commissioned only the 25 and 30 mm UICs.

**TABLE 1:** Component list for the major components of the UIC system.

Description	Part #	Description	Part #
UIC, 25 mm	GM1011013310	plastic interstitial needle, sharp tip	GM11007580
UIC, 30 mm	GM1011013320		
UIC, 35 mm	GM1011013330	plastic interstitial needle, blunt tip	GM11010750
UIC, 40 mm	GM1011013340		

Description	Part #	Description	Part #
UCP, 40 mm, straight	GM11011540	UCP, 30 mm, straight	GM11011510
UCP, 40 mm, curved 15 degree	GM11011560	UCP, 30 mm, curved 15 degree	GM11011520
UCP, 40 mm curved 30 degree	GM11011560	UCP, 30 mm curved 30 degree	GM11011530
UCP, 60 mm, straight	GM11011600	UCP, 50 mm, straight	GM11011570
UCP, 60 mm, curved 15 degree	GM11011610	UCP, 50 mm, curved 15 degree	GM11011580
UCP, 60 mm curved 30 degree	GM11011620	UCP, 50 mm curved 30 degree	GM11011590
UCP, 80 mm, straight	GM11011660	UCP, 70 mm, straight	GM11011630
UCP, 80 mm, curved 15 degree	GM11011670	UCP, 70 mm, curved 15 degree	GM11011640
UCP, 80 mm curved 30 degree	GM11011680	UCP, 70 mm curved 30 degree	GM11011650

*Note*: Component list for the major components of the UIC system – Universal Interstitial Cylinder (UIC), plastic interstitial needles, and both the even and the odd lengths sets of the plastic Universal Cervix Probes (UCP). Other components necessary for use of the UIC system may be found in Varian's instruction for use (IFU) documentation.

AAPM Task Groups 56 and 59^1^, ^2^ as well as similar international groups^3^, ^4^, ^5^ recommend that we, as the end user, must perform commissioning of new applicators to guarantee the proper and safe delivery of the intended dose. In this paper, based on the AAPM guidelines for brachytherapy applicators and our own clinical experience, a set of tests for the commissioning of the UIC system is presented. These include the tests described in Table 5 of Section 7 in the AAPM medical physics practice guideline 13.a: HDR brachytherapy^6^, which outlines hardware QA commissioning requirements including those for multi‐use applicators, as well as clinical implementation tests using 3D imaging modalities. With the current importance of MR imaging as well as CT imaging for brachytherapy target delineation and treatment planning,^7^, ^8^ it was necessary to design a unique phantom to minimize the introduction of water‐like liquid into the needle guide tracks, and the investigation of various marker strands to determine which provided the clearest and most accurate reconstruction of needle locations and therefore source positions. This institution‐based creation of unique phantoms for use in applicator commissioning with MR modalities is seen in other cases, as well.^9^, ^10^, ^11^, ^12^, ^13^

## 2 METHODS

### 2.1 Integrity and dimension check

All components of the UIC system were evaluated visually and with mega‐voltage (MV) imaging. First, visual inspections were performed to assess structural integrity and to make physical measurements where beneficial, such as in the case of the overall length of the sharply pointed interstitial needles where the exact tip of the needle was hard to determine on imaging. Second, a more robust verification of integrity was made of all components by taking 2D images using the 2.5 MV (high quality protocol) imaging available on the Varian TrueBeam linear accelerators. The applicator was placed at isocenter (i.e., magnification factor of 1) to accurately measure the dimensions of the applicator. These images were also used to verify several applicator dimensions for the UICs, UCPs, and interstitial needles. For the UICs, the outer diameters of the UIC shell and core were measured and compared to nominal values specified by the manufacturer. The length of the UIC components were also measured and compared to other UICs of the same nominal size. For the UCPs, the MV images were used to measure the total length of the probe and the probe length past the flange and these values were compared to the manufacturer's nominal values. For the interstitial needles, the MV images and a physical ruler were used to measure the overall length of the needle. While it is possible to measure certain dimensions manually, such as the UIC diameters with calipers and the straight UCP lengths with a ruler, it was more accurate and consistent to measure the angled UCPs and all internal dimensions using the 2D MV images.

### 2.2 HDR ^192^Ir source offset determination from the tip of the applicator

For all the UCPs, kilo‐voltage (kV) images (extremity, medium protocol) were taken using the imaging available on the Varian TrueBeam linear accelerators with VMS‐numbered markers (“dummy strands”) inserted to the distal end of the applicators. These images were used to determine the distance from the outside of the applicator tip to the middle of the first dwell position (“offset”). Compared to the MV imaging, the kV imaging has higher image contrast to distinguish the radio‐opaque marker from the plastic applicator part. For the UCPs, this clinical offset measurement was compared to the nominal offset measurement provided by the solid applicator (SA) model in the VMS BrachyVision (BV) Treatment Planning System (TPS). Note that the rigid guide tube, to be used in the center channel of the UIC when a cervix probe is not indicated, had already been commissioned and in use in our clinic with the VMS segmented cylinder set and so is not addressed in this paper.

Autoradiographs were also performed on a subset of UCPs using Gafchromic EBT3 films (Ashland Specialty Ingredients, New Jersey, USA). The applicators were taped to the Gafchromic film and the physical end of the applicators were marked on the film. The applicators were connected to the GMPiX afterloader using appropriate source guide tubes (SGTs) and dwell times were selected to produce a radiograph of adequate density on the film. After radiation exposure, the films were scanned on an Expression 11000XL flatbed scanner (Epson America, Inc., California, USA) with a resolution of 150 × 150 dpi and line profiles were drawn in RIT software, version 6.3 (Radiological Imaging Technology, Inc., Colorado, USA) to measure the distance between the physical end of the applicator as marked on the film and the center of the first dwell.

### 2.3 Channel clearance and overall length check

For all the UCPs and all the interstitial needles, the channel clearance and source‐to‐indexer distance and reproducibility was evaluated using two methods. For both methods, the appropriate SGTs were attached to the applicators. Both the interstitial needles and the UCPs are used with the black click‐fit SGTs (98 cm in length) for use with 32 cm applicators. In the first method, the overall length and channel clearance of each component and SGT combination was checked manually using the VMS 130 cm length gauge. The other method is to check the length and channel clearance mechanically via a length test performed by attaching the SGTs to the GMPiX afterloader and initiating a plan that ran the source to each applicator. The “check all, check each” setting was used and it performed two dummy checks and a source wire check on each applicator.

### 2.4 Treatment planning test for a clinical case: One UIC applicator set

**FIGURE 1:**
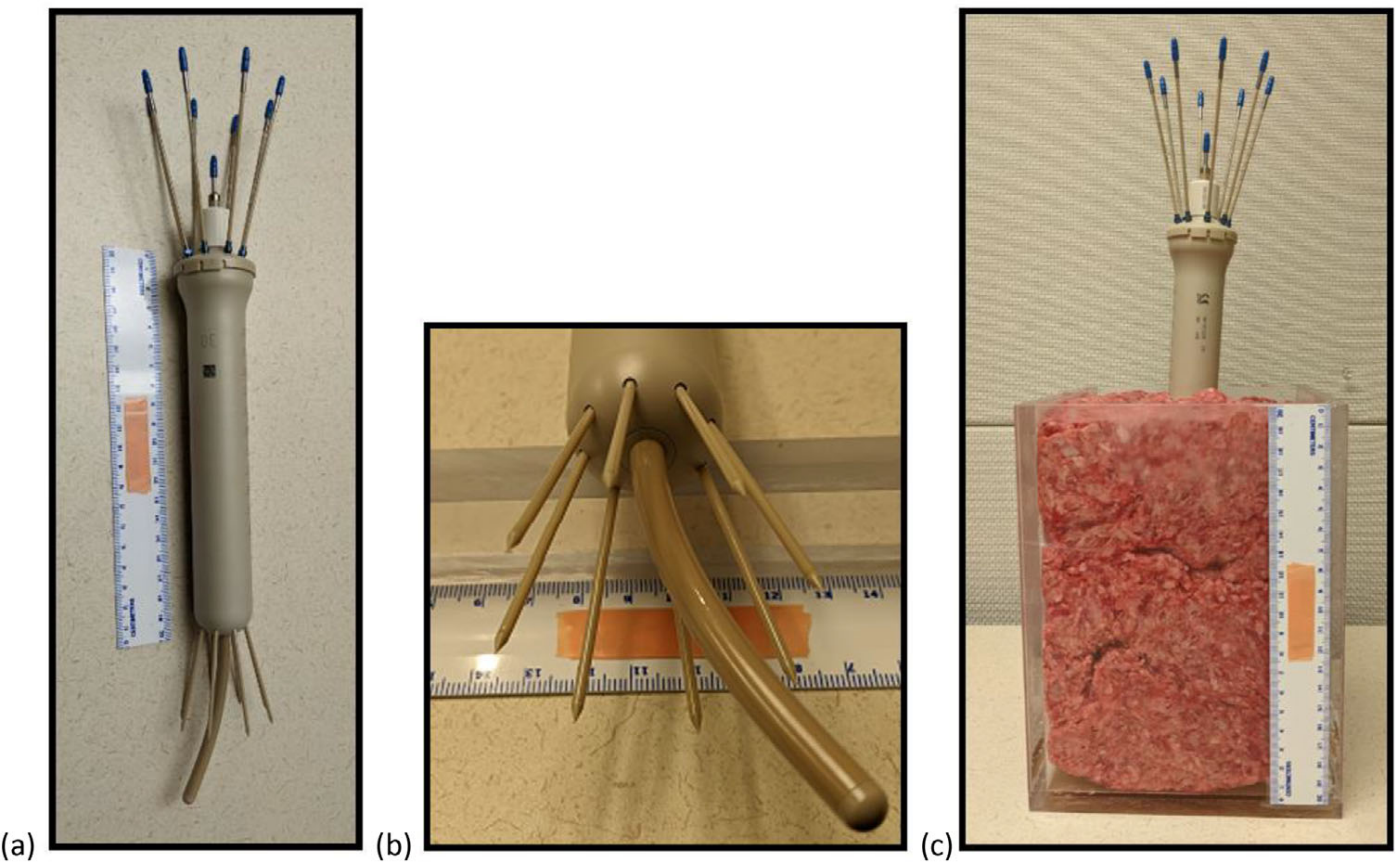
The UIC components used for clinical implementation tests. Panels (a) and (b) show the combination of UIC components used for clinical implementation tests. These were the UIC with 30 mm diameter and the plastic UCP with length beyond flange of 80 mm and probe angle of 30‐degrees in the center channel #1, and eight plastic interstitial needles inserted into UIC channels #2 through #9. Panel (c) shows the phantom made of ground beef that was constructed for the clinical implementation tests of the UIC applicator set. The phantom containing the applicator set was imaged using both CT and MR modalities. UCP, universal cervix probe; UIC, universal interstitial cylinder.

A treatment planning test for the clinical implementation was performed using one specific combination of UIC, UCP, and eight needles in a phantom to simulate a patient implant. The specific combination of components used was the 30 mm diameter cylinder UIC, the 80 mm length beyond flange, 30‐degree probe angle plastic UCP, and eight plastic interstitial needles inserted into UIC channels #2 through #9. See Figure 1a,b for this assembled UIC combination.

#### 2.4.1 Phantom construction to mimic a clinical case

Three gynecological phantoms were constructed with one of three different phantom materials: water, gelatin, or ground beef. The water phantom was made of towels soaked in water and packed around the applicator set. The gelatin phantom was constructed by mixing a batch of food‐grade gelatin and then stabilizing the applicator in the gelatin until it set. The ground beef phantom was made of 80% lean, 20% fat ground beef packed into a container around the applicator set. Figure 1c shows this ground beef phantom. After being placed in each of these three different gynecological phantoms, both CT and MR scans were taken. The CT scans were taken on a Siemens Biograph mCT scanner (Siemens Medical Solutions USA, Inc. Knoxville, TN) with a slice thickness of 0.6 mm. The MR scans were taken on a 3T Magnetom Skra (Siemens) using the following clinical HDR GYN MRI clinical imaging protocol scans using body coil: T1w – 3d spgr 1.0 mm isotropic (TR = 3.7, TE = 1.3), FOV 300 × 300, and T2w – axial 3.0 mm (TR = 2000, TE = 121), FOV 300 × 300. These CT and MR scans were imported into BV and their image quality for treatment planning was investigated.

#### 2.4.2 Channel identification and source location determination

Three different methods for channel identification were investigated to determine which method allowed the most accurate determination of needle location and therefore source positions. These methods were as follows: (1) no radio opaque markers, (2) VMS‐numbered markers (“dummy strands”), (3) nylon coated stainless steel leader wires (“fish wire”). Channel identification was performed on CT images using these three different methods. The ability to see the needles within the needle guide track, as well as any resulting artifact from the radio opaque markers was assessed. An additional MR marker for use during the MR scans was investigated. An HDR MRI Lumen Marker (C4 imaging, LLC, Doylestown, PA) was placed in the needle in one of the outer channels (channel #9) prior to the MR scan. The MR scans were then assessed to see if the MR marker was visible enough to check for any applicator rotation and confirm orientation for the CT/MR registration required prior to treatment planning.

#### 2.4.3 Validation of SA models in BrachyVision

In order to expedite the treatment planning using the UIC applicator, the vendor provides an solid applicator (SA) model in the TPS. The dimensions from these vendor‐provided SA models are based on the computer‐aided design model files of the specific applicator so measurements made of these SA models can be considered to be nominal vendor‐provided dimensions. All available SA models for the UIC components of interest, which included both UIC diameters and all the UCPs, were imported into the local BV SA library. Each of these applicators was imported into a virtual water phantom image set in BV and dimensions of all components of the applicator were measured using a digital ruler tool within BV. For all the UIC and UCPs under investigation, measurements were made on their respective SA models to confirm they agreed with their physical dimensions. For the UIC this included diameter and length and the distance from the outer surface of the UIC to the inside of the needle guide track and to the center of the needle. For each of the UCPs, the probe angle, length past the flange, and offset was measured on the SA model and compared with physical measurements. In addition, the specific components used in the clinical implementation test were overlaid on the 3D planning CT scan to confirm these measurements against a physical applicator and also to confirm their coincidence with the physical channels for both the UIC and the UCP used.

## 3 RESULTS

### 3.1 Integrity and dimension check

Visual inspections of the UIC components did not show any obvious structural defects, fractures, or issues with integrity. The digital radiographic images also confirmed the absence of any obvious structural defects.

**FIGURE 2:**
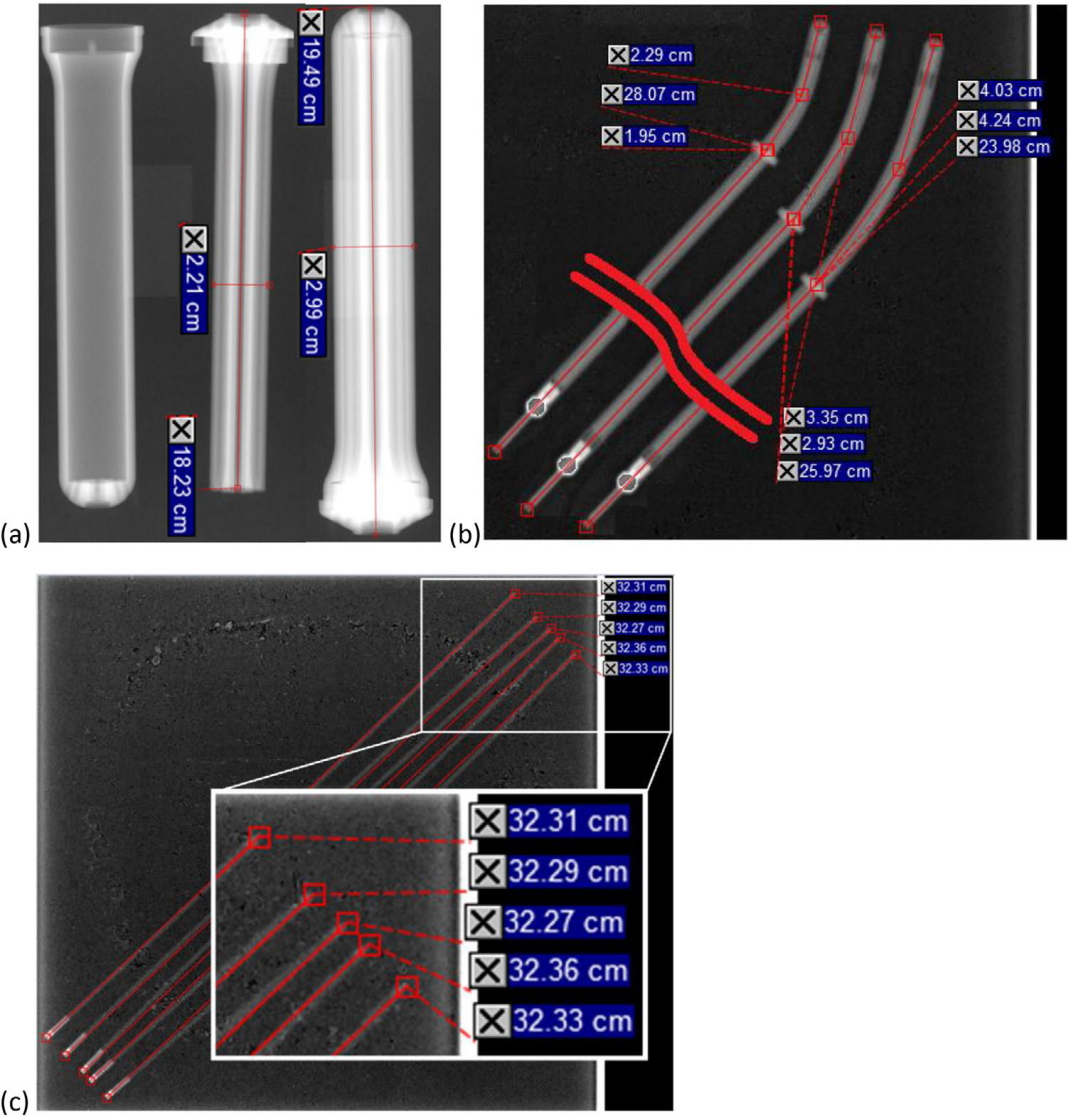
MV images of UIC components. 2D MV images were taken of all components to confirm measurements and dimensions as well as to look for any structural defects. Panel (a) shows the measurements made of the 30 mm UIC, (b) shows measurements made of the 30 degree, even length, UCP set, and (c) shows measurements made of five plastic interstitial needles. When these images were examined for structural defects, none were found during commissioning. MV, mega‐voltage; UCP, universal cervix probe; UIC, universal interstitial cylinder.

Table 2 lists measurements from the 25 and 30 mm UICs. Two UICs of each diameter were commissioned and all measurements are included. The nominal outer diameter of the UIC shell is given in the Instruction For Use (IFU) and is either 25 or 30 mm, and the nominal diameter of the UIC core is the nominal outer diameter of the shell minus approximately 7.0 mm to account for the thickness of the UIC shell wall. MV‐image based measurements of the shell diameters were all within 0.1 mm and measurements of the inner core diameters were within 0.9 mm. Overall lengths of the assembled UIC applicator and the lengths of the UIC cores were measured for inter‐comparison, but no nominal vendor‐supplied value is available. Lengths of the assembled UIC measured 194.9 ± 0.4 mm and lengths of the UIC cores measured 182.7 ± 0.3 mm. Figure 2a shows the 2D MV image of one of the 30 mm UICs and the measurements of overall UIC diameter and length as well as the diameter and length of the core of the UIC.

**TABLE 2:** Measurements from the commissioned 25 and 30 mm UICs made using the 2D MV images.

UIC shell			UIC core		
Nominal diameter (mm)	Measured diameter (mm)	Assembled measured length (mm)	Nominal diameter (mm)	Measured diameter (mm)	Measured length (mm)
25	25.0	194.7	18	17.7	182.9
25	24.9	194.5	18	18.1	183.0
30	29.9	194.9	23	22.1	182.3
30	29.8	195.4	23	22.8	182.7

Abbreviations: MV, mega‐voltage; UIC, universal interstitial cylinder.

**FIGURE 3:**
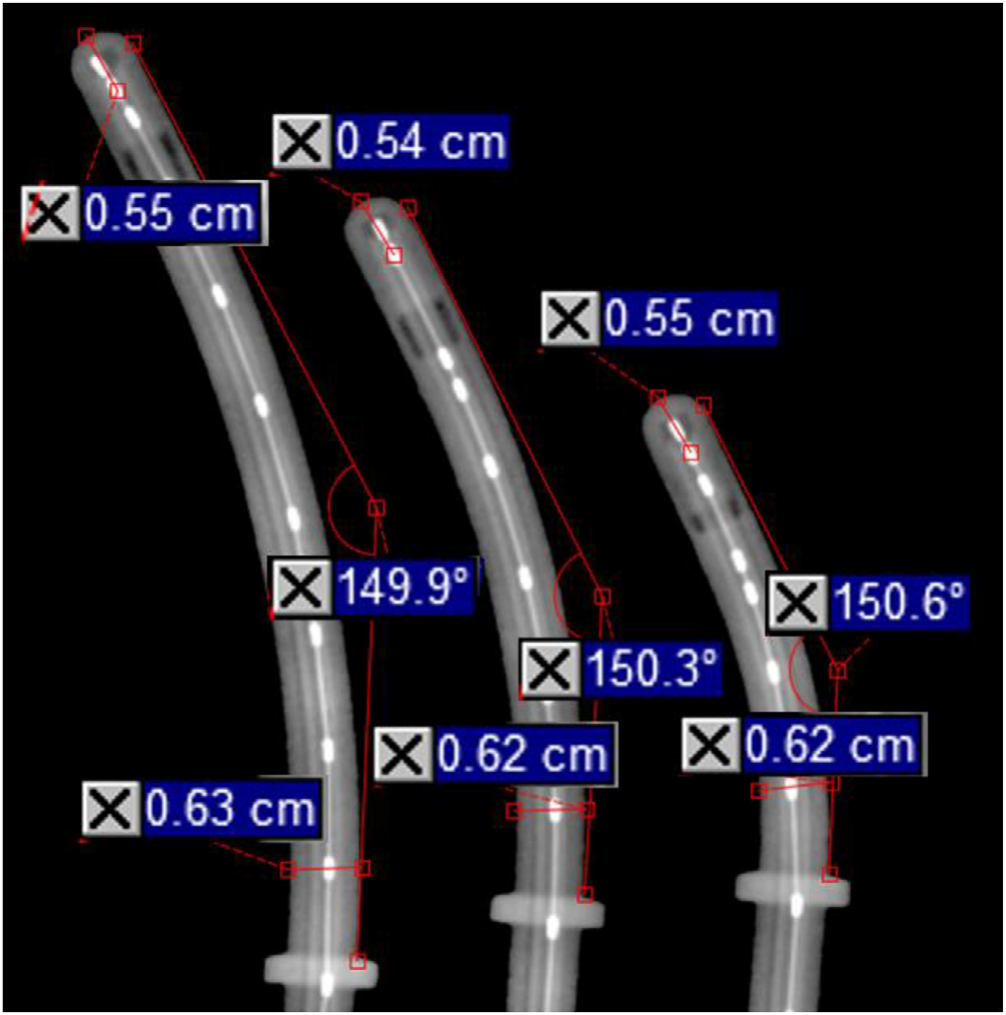
kV images of UCPs taken with VMS markers. After inserting a VMS‐numbered marker strand into each applicator, 2D kV images were taken of all UCPs. On these images, additional measurements were taken and the distance from the outside of the applicator to the center of the first dwell (“offset”) for each applicator was determined. Shown here are the 2D kV images of the 30 degree, even length UCP set with applicator angle, applicator diameter, and source offset measured. For the UCPs, the nominal diameter of 6.3 mm was from the VMS IFU and the nominal offset of 5.5 mm were determined from the SA model. IFU, instruction for use; kV, kilo‐voltage; UCPs, universal cervix probes; VMS, Varian Medical Systems.

Table 3 lists measurements from both available sets of UCPs – those with even number lengths (40, 60, and 80 mm) and odd number lengths (30, 50, and 70 mm). As per the VMS IFU for the UIC set, the thickness of the wall of the UCP is 2.2 mm, so with an inner channel length of 320.0 mm, the nominal overall length is 322.2 mm and the measured length of the probe past the flange should be 2.2 mm greater than the nominal stated length past the flange. Figure 2b shows the 2D MV image of the 30 degree, even length UCP set and illustrates the measurements of the overall probe length and the length of the probe past the flange. All measurements based on 2D MV imaging agreed with the nominal length to within 1.4 mm or 1 degree. Table 3 also lists measurements made from 2D kV images that were taken with VMS‐numbered strands and analyzed to determine the angle of the probe and diameter of the applicator. Figure 3 show the 2D kV images of the 30 degree, even length UCP set and the associated measurements. The nominal diameter of 6.3 mm is from the VMS IFU.

**TABLE 3:** Measurements from both even and odd sets of the UCPs.

Listed probe angle and length past flange	Nominal length past flange (mm)	MV‐measured length past flange (mm)	Nominal overall Length (mm)	MV‐measured overall length (mm)	Nominal probe angle	kV‐measured probe angle	Nominal diameter (mm)	kV‐measured diameter (mm)
15°, 40 mm	42.2	42.6	322.2	323.3	15°	14.3°	6.3	6.2
15°, 60 mm	62.2	62.3	322.2	323.2	15°	14.9°	6.3	6.3
15°, 80 mm	82.2	82.6	322.2	323.6	15°	14.8°	6.3	6.2
30°, 40 mm	42.2	42.4	322.2	323.1	30°	29.6°	6.3	6.2
30°, 60 mm	62.2	62.5	322.2	322.5	30°	29.7°	6.3	6.2
30°, 80 mm	82.2	82.3	322.2	322.5	30°	30.1°	6.3	6.3
0°, 40 mm	42.2	42.4	322.2	323.0	0°	–	6.3	6.2
0°, 60 mm	62.2	62.3	322.2	323.1	0°	–	6.3	6.2
0°, 80 mm	82.2	82.5	322.2	322.8	0°	–	6.3	6.2
15°, 30 mm	32.2	32.3	322.2	323.2	15°	14.3°	6.3	6.3
15°, 50 mm	52.2	52.4	322.2	322.9	15°	14.9°	6.3	6.1
15°, 70 mm	72.2	72.2	322.2	324.2	15°	14.9°	6.3	6.3
30°, 30 mm	32.2	32.2	322.2	323.9	30°	28.5°	6.3	6.3
30°, 50 mm	52.2	52.3	322.2	323.1	30°	29.7°	6.3	6.3
30°, 70 mm	72.2	72.4	322.2	322.8	30°	29.0°	6.3	6.3
0°, 30 mm	32.2	32.3	322.2	323.5	0°	–	6.3	6.3
0°, 50 mm	52.2	52.4	322.2	323.5	0°	–	6.3	6.3
0°, 70 mm	72.2	72.4	322.2	323.5	0°	–	6.3	6.5

*Note*: Measurements from both the even and odd sets of the UCPs. Columns to the left were measured from MV images and columns to the right were measured from kV images. Total probe length, length past flange, probe angle, and probe diameter are compared to manufacturer nominal values. Measured length past flange includes the 2.2 mm thickness of the UCP wall so the nominal value for comparison is the nominal 320 mm plus an additional 2.2 mm.

Abbreviations: kV, kilo‐voltage; MV, mega‐voltage; UCP, universal cervix probe.

Figure 2c shows the 2D MV images of five of the plastic interstitial needles initially commissioned and their associated length measurement. As per the VMS IFU, the nominal physical length of the pointed tip of the sharp interstitial needle is approximately 4.6 mm, so for an inner channel length of 320 mm, the measured external length of the needles should be approximately 324.6 mm. Table 4 lists the measurements made on five of the interstitial needles using MV images and shows measured needle lengths to be within 2 mm of the expected nominal value with all needles measuring slightly shorter. This variation and its direction resulted from the difficulty in visualizing the exact tip of a highly pointed needle on the MV image. Measurements of the needles with a physical ruler avoided this difficulty, and those measurements were within 0.2 mm of the nominal value and measured both shorter and longer than the nominal value.

**TABLE 4:** Measurements made on five of the interstitial needles using MV images and a physical ruler.

Needle number	Nominal length (mm)	MV‐measured length (mm)	Physical ruler‐measured length (mm)
#1	324.6	323.1	324.5
#2	324.6	322.9	324.7
#3	324.6	322.7	324.4
#4	324.6	323.6	324.5
#5	324.6	323.3	324.6

### 3.2 HDR ^192^Ir source offset determination from the tip of the applicator

The source offset of 5.5 mm from the physical end of the UCP to the center of the first dwell position was determined from the SA model and the measurements made on the UCPs from the kV images are included in Table 5. All measurements were within 0.3 mm of the nominal value.

**TABLE 5:** Measurements of source offset made on UCPs based on kV images and autoradiographs.

Nominal probe angle	Nominal length past flange (mm)	kV‐measured source offset (mm)	Autoradio graph measured source offset (mm)
15°	40	5.4	–
15°	60	5.5	6.0
15°	80	5.5	–
30°	40	5.5	–
30°	60	5.4	–
30°	80	5.2	5.0
0°	40	5.3	5.5
0°	60	5.4	–
0°	80	5.3	–
15°	30	5.2	–
15°	50	5.5	5.8
15°	70	5.5	–
30°	30	5.3	–
30°	50	5.3	–
30°	70	5.6	5.8
0°	30	5.4	5.5
0°	50	5.5	–
0°	70	5.3	–

*Note*: Commissioning of the Varian universal interstitial cylinder system.

Abbreviations: kV, kilo‐voltage; UCP, universal cervix probe.

**FIGURE 4:**
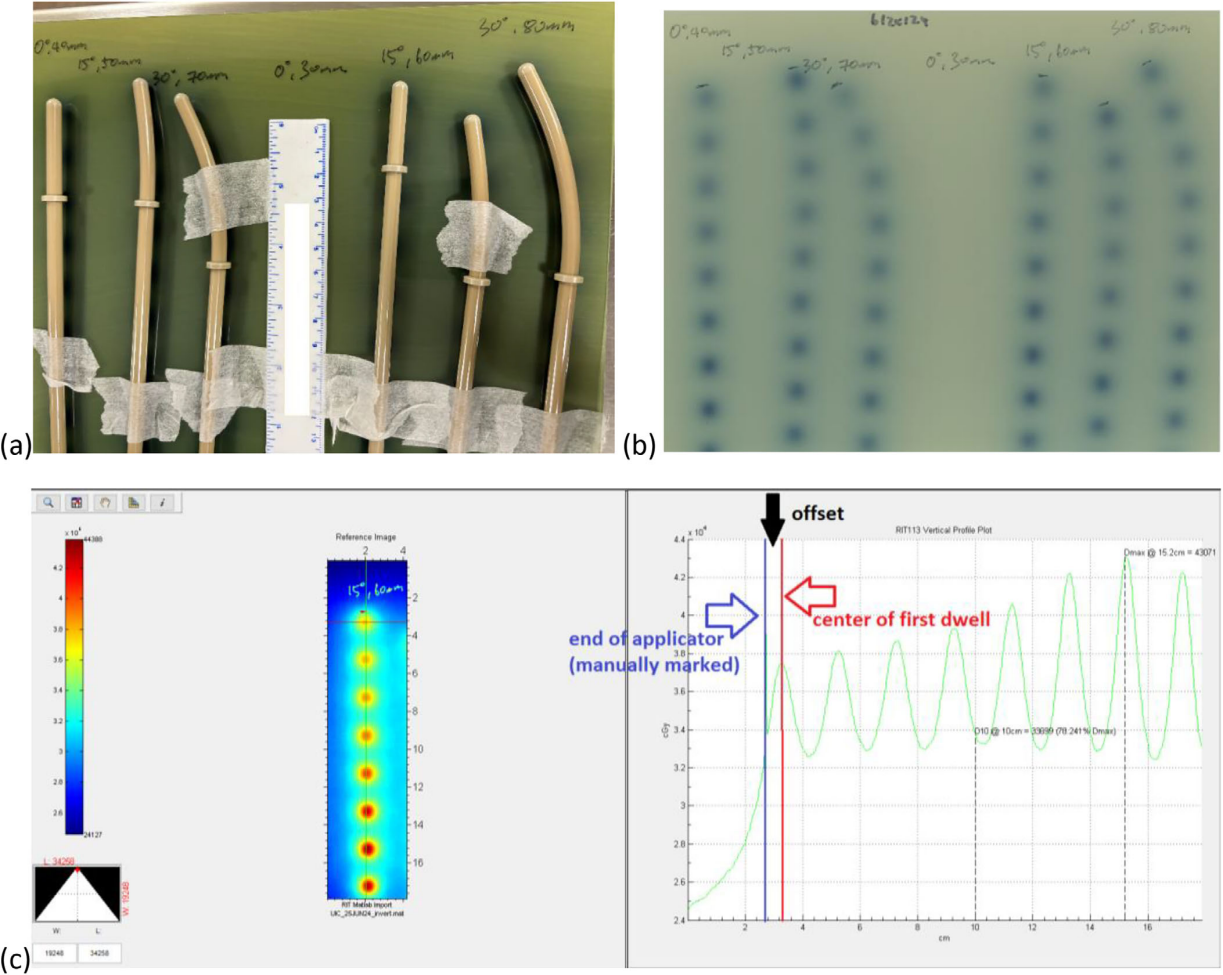
Autoradiographs used to determine source offset. Panel (a) shows the arrangement of the UCPs on the Gafchromic film after irradiation. Panel (b) shows the resulting autoradiograph of the setup in panel (a). Panel (c) shows the associated scan and the offset measurement of the 15 degree, 60 mm length UCP using the end of the applicator as marked on the film and the center of the first dwell as determined by the maximum pixel value. UCP, universal cervix probe.

The autoradiographs and associated scans are shown in Figure 4a–c. Measurements of the source offset of the UCPs for the included subset of applicators are included in Table 5 and all measurements are within 0.5 mm of the nominal value.

### 3.3 Channel clearance and overall length check

The results of the channel clearance and source‐to‐indexer distance and reproducibility using both methods were evaluated on a pass/fail basis. All SGTs in routine clinical use are checked during clinical quarterly QA following Ir‐192 source exchange to ensure they are the correct length and they maintain their functionality. When these SGTs of the correct length and type were used with the plastic interstitial needles and both the even and odd length sets of UCPs, all components passed both the manual length/clearance check using the length gauge and also passed the mechanical length test when connected to the HDR afterloader (GammaMedPlus iX) using a test plan initiated at the console.

### 3.4 Treatment planning test for a clinical case: One UIC applicator set

#### 3.4.1 Phantom construction to mimic a clinical case

Using water as phantom to mimic patient environment is natural choice but comes with a critical limitation in this case. When the combination of UIC components was assembled and placed into a liquid‐water based phantom consisting of water‐soaked wet towels and then imaged, it quickly became clear that there was an issue with unwanted signal from the channels for interstitial needles on both the CT and MR scans. This unintentional signal resulted from water infiltrating the needle track guides from the distal end of the UIC. To allow insertion of the needles through the UIC channels, the openings in the UIC must be larger than the diameter (i.e., 2 mm) of the needles as shown in Figure 1b. Because of these openings, with or without needles inserted into the guide tracks, the phantom made of wet towels allowed significant amounts of liquid to enter the needle guide tracks from the holes in the distal end of the UIC.

**FIGURE 5:**
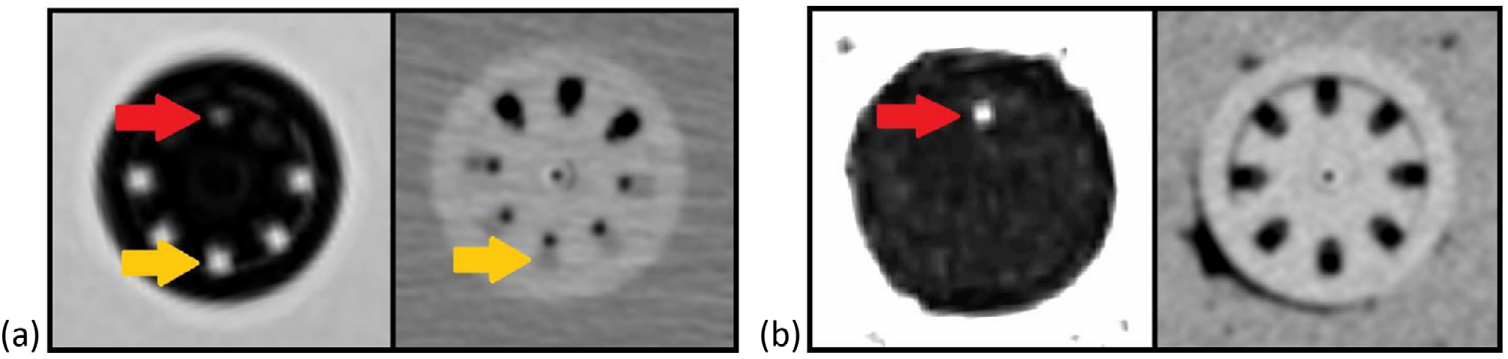
Fluid in needle guide tracks and MR marker visibility. Unintended signal due to fluid in the needle guide tracks is indicated by the yellow arrows. The red arrow indicates the MR marker that was also investigated. Panel (a) shows T1 weighted MR image (left) and CT image (right) from the gelatin phantom with this unintended signal, (b) shows T1 weighted MR image (left, window leveled to highlight the MR marker) and CT image (right) from the ground beef phantom which demonstrated that the use of this type of phantom successfully limited the introduction of liquid into the UIC needle guide tracks. UIC, universal interstitial cylinder.

A gelatin phantom was constructed to try to reduce or eliminate the amount of liquid in the channels, but as the applicator had to be placed in the phantom before the gelatin hardened, the liquid still infiltrated the needle guide tracks and led to unintended MR and CT signal. Figure 5a shows the extent of this unintended signal on both CT and MR scans for the gelatin phantom.

The construction and use of a ground beef phantom limited the introduction of liquid into the UIC needle guide tracks. Figure 5b shows the extent to which this liquid was eliminated from the channels when scans were taken with the ground beef phantom. As seen in the sample scans, the packing of the ground beef around the UIC applicator set was imperfect, but even with appreciable air pockets around the applicator, the CT and MR image quality remained clinically useful for treatment planning, and channel identification and digitization was greatly simplified without the unwanted CT and MR signal from the liquid infiltration into the needle guide tracks. Hence, scans performed using the ground beef phantom were considered to be closest to those from a clinical case.

**FIGURE 6:**
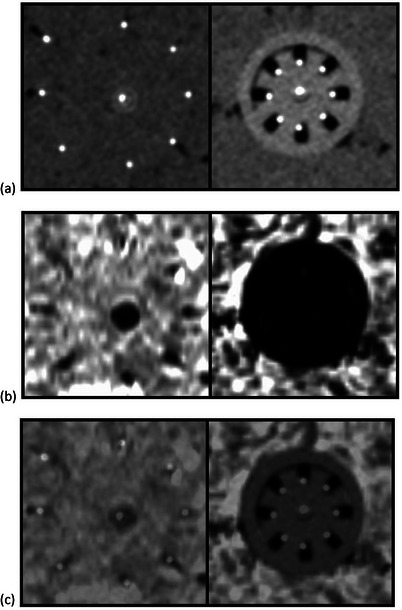
CT/MR image registration. Shown here are cross sections focusing on either the interstitial needles or the UIC body after CT/MR image registration. Panel (a) shows the CT image at a slice showing either the needles in tissue (left figure) or the applicator body (right figure), (b) shows the MR image at the same level (tissue in left and applicator body in right) after CT/MR registration, and (c) shows a 50/50 blend between the CT and MR images. UIC, universal interstitial cylinder.

The CT and MR scans taken with the ground beef phantom were used for treatment planning practice, and were used both to investigate their image quality for CT/MR image registration as well as to determine how to digitize the plastic interstitial needles. Figure 6a–c show the coincidence between the cross sections of the interstitial needles or the UIC body after image registration.

#### 3.4.2 Channel identification and source location determination

**FIGURE 7:**
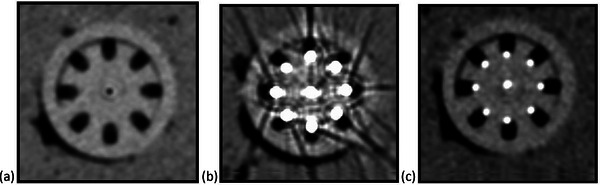
Use of radio opaque markers to identify needle location. Various methods of identifying the needle location, and therefore the source position within the needles, for applicator digitization on CT scans are shown here. Panel (a) shows the CT scan with no markers in the needles, (b) shows the CT artifacts present when using the VMS‐numbered markers, and (c) shows a CT scan using a fish wire marker in each channel. VMS, Varian Medical Systems.

The plastic interstitial needles are difficult to visualize in the CT scans without the use of any markers, and CT images with no markers display substantially wider needle guide tracks than the plastic needles as shown in Figure 7a. When CT scans were taken using VMS‐numbered marker wires, the markers resulted in significant CT artifacts as shown in Figure 7b. CT scans taken with the fish wire inserted into the interstitial needles provided the best visibility of the needles within the needle guide tracks as shown in Figure 7c. The use of an MR marker allowed the selected needle, channel #9 in this case, to be identified on the T1 weighted MR scan as indicated by the red arrows in Figure 5.

**FIGURE 8:**
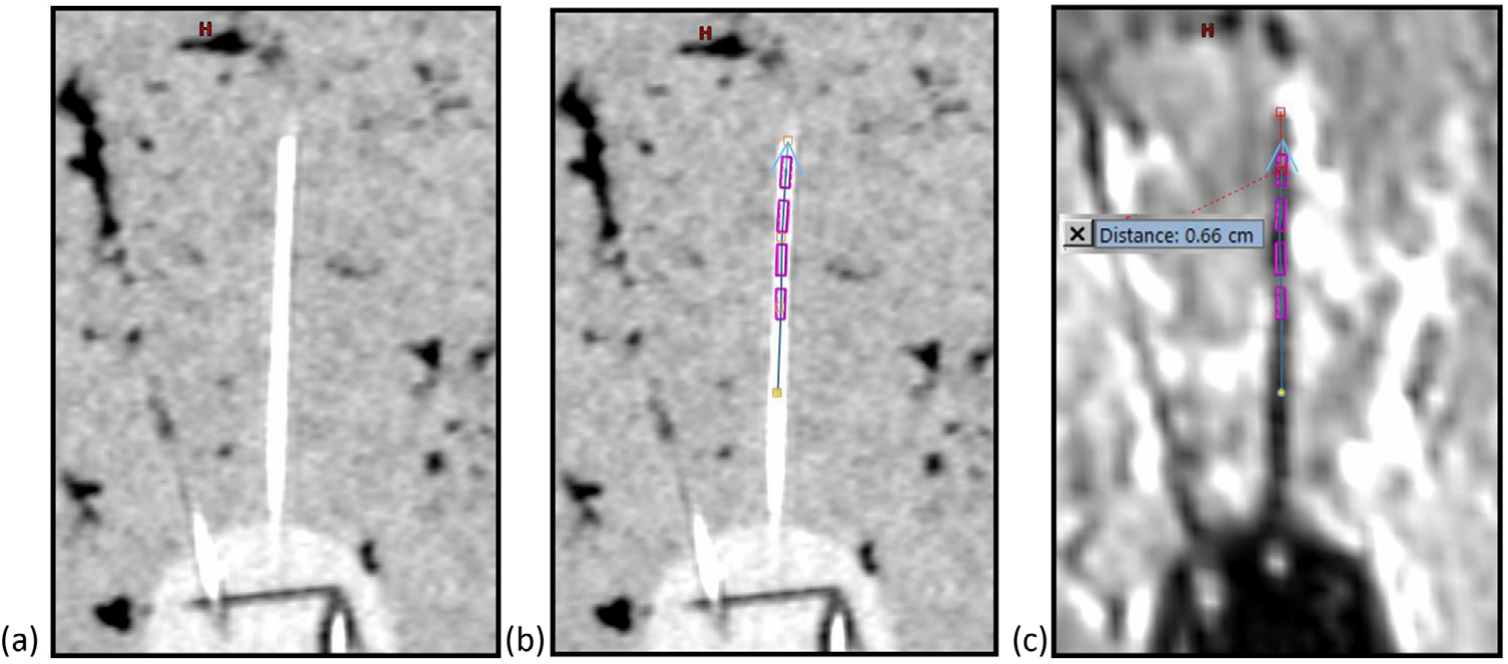
Signal void on MR images. After CT/MR image registration, an MR signal void is seen past the end of the digitized needle due to the physical dimension of the needle. Panel (a) shows an interstitial needle containing fish wire on CT image, (b) shows the digitized needle with the beginning of the needle placed at the end of the visible fish wire on CT images, and (c) shows the signal void beyond the tip of the digitized needle on T1 weighted MR image.

Because the fish wire is pushed to the end of the needle, the signal from the fish wire extends to the tip of the inside cavity of the needle, so when digitizing the needle on CT images, the digital tip of the needle is placed at the furthest visible extent of the fish wire, as shown in Figure 8a,b.

When blended with properly registered T1 weighted MR images, a signal void is seen beyond the tip of the digitized applicator due to the physical material of the plastic needle, as shown in Figure 8c. The overall distance between the end of the MR signal void and the center of the first dwell as seen in the digitized applicator is approximately 6.6–7.0 mm and consists of the MR signal void resulting from the combination of the pointed plastic tip of the needle (4.6 mm as per the VMS IFU) and the inherent offset from the inner wall of the needle tip to the middle of the first dwell position (3.5 mm).

#### 3.4.3 Validation of SA models

**FIGURE 9:**
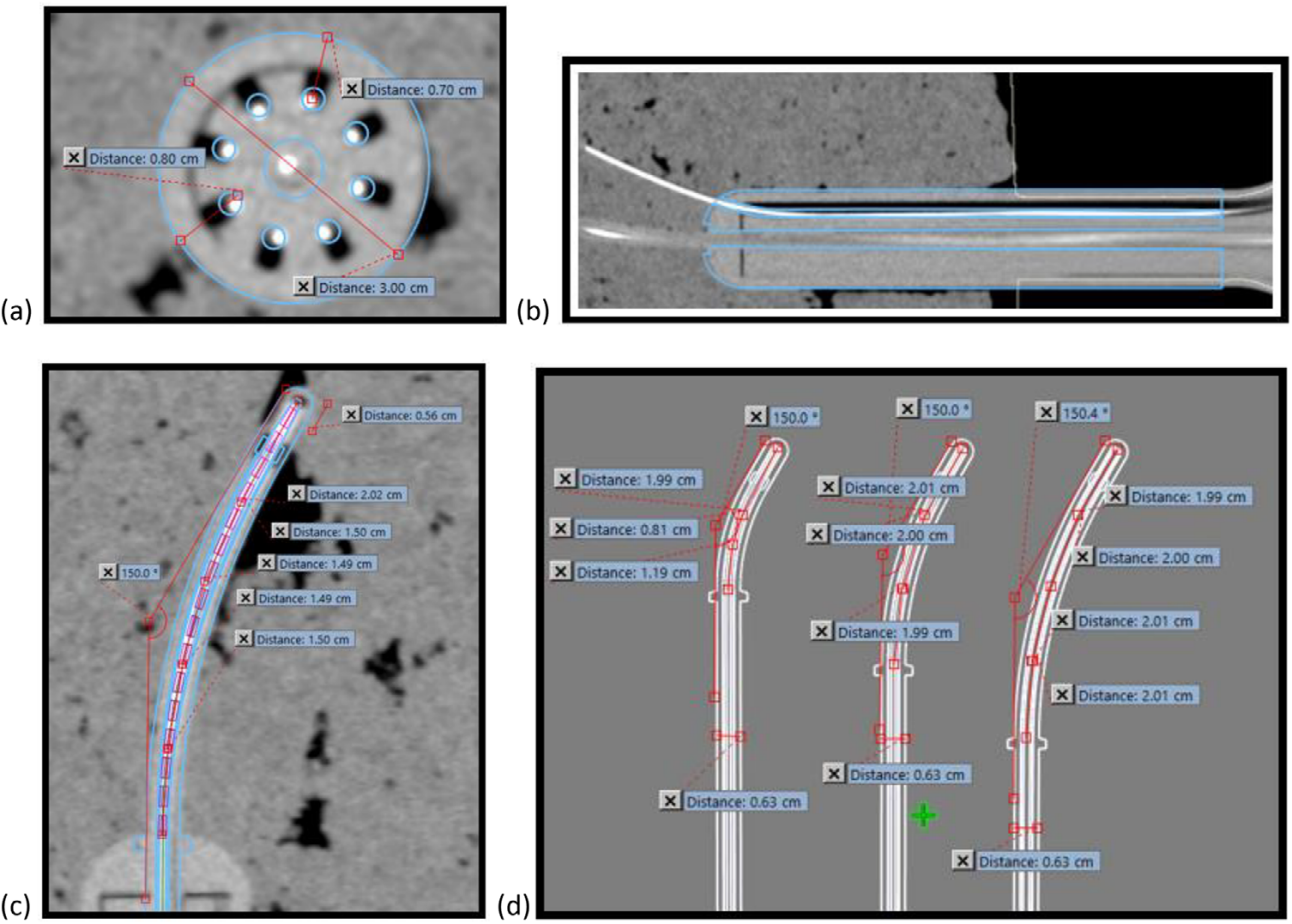
SA models. Shown here are the SA models for the UIC and the UCP sets imported into a CT image set of the UIC/UCP assembly embedded in the ground beef phantom (Panels a–c). Panel (a) shows UIC SA measurements and coincidence with the interstitial needles, (b) shows the coincidence of the interstitial needles with the UIC channels along the length of the channels, (c) demonstrates source channel coincidence between the SA and the scan of the UCP, and (d) shows the measurements of the 30 degree, even length, UCP set made to validate the SA model. For the measurements in Panel (d), the applicators were imported into a virtual water phantom and the applicator length past flange was determined to the inner wall. SA, solid applicator; UCP, universal cervix probe; UIC, universal interstitial cylinder.

This fish wire scan that most clearly showed the needle paths was used along with the UIC SA model to confirm coincidence of the source channels and also to confirm the distance from the outer wall of the UIC to the inside of the needle guide track (8.0 mm) and to the center of the 2.0 mm diameter interstitial needle (7.0 mm) as well as the 30 mm diameter of the cylinder as shown in Figure 9a. The coincidence of the needle guide tracks and the interstitial needles inserted into those tracks extends along the entire length of the UIC, as shown in Figure 9b.

After being imported into the CT image set, the SA model for the appropriate UCP was investigated for source channel coincidence and also measured for probe length beyond the flange, probe diameter, probe angle, and source offset, and was compared to the physical applicator on the CT image. Figure 9c shows source channel coincidence. Figure 9d shows the measurements of the 30‐degree, even length UCP set. For the 30‐degree probes shown, the nominal probe angle and diameter matched within 0.6 degrees and 0.0 mm, respectively. Lengths beyond the flange for the 40, 60, and 80 mm UCPs, when measured to the interior wall rather than the outer wall with its 2.2 mm thickness, as was done in Section 3.1, were 39.9, 60.0, and 80.1 mm, respectively. All other SA measurements made on the remaining UCP sets compared similarly and as the SA commissioning was considered to be pass/fail if nominal angle was within 1 degree and other measurements were within 1 mm, all SA models were considered to have passed.

## 4 DISCUSSION

In our clinic, the UIC has been used for both locally advanced cervical cancers with substantial vaginal extension as well as primary vaginal cancers. The multi‐channel option allows for improved organs‐at‐risk sparing over single‐channel cylinders in clinical cases where there is vaginal disease laterality and/or tumor thickness >5 mm. Several publications have highlighted the versatility of multi‐channel cylinders, particularly in combination with interstitial needles as part of image‐based brachytherapy.^14^, ^15^, ^16^, ^17^ Hybrid applicators such as the UIC allow for a faster and less invasive alternative to traditional template‐based interstitial brachytherapy.

With any new applicator, there are new challenges: The use of plastic components may make digitization difficult in certain clinical cases. Although every attempt was made to simulate clinical conditions, there were still some challenges when the UIC system was used clinically and there are additional steps that can be taken to ensure the safe treatment using the UIC, though this requires increased allowances for planning time with the UIC.

One issue with the use of flexible plastic needles and the design of the UIC is that the wide needle guide tracks introduce a level of uncertainty as to the exact location of the needles within the tracks and therefore the source positions within the applicator. For this reason, it is especially important to use the SA model and some sort of radio‐opaque marker that allows accurate determination of the position of the interstitial needles within the needle guide tracks. The use of some sort of marker within the channel of the needle is also vital to determining the end of the needle and therefore the location of the first dwell position, as the standard treatment planning practice of measuring a specific source offset value from the visible end of the applicator cannot be utilized. Instead, since the sharp tip of the plastic needle cannot be seen explicitly in either 2D or 3D images, the tip of the digital applicator should be placed at the most distal portion of the needle insert (fish wire or any other type of marker) that can be seen. The MR void shown in Figure 8c confirms this technique. This 6.6 to 7.0 mm void seen in the in‐phantom scans is slightly less than the nominal physical distance from the needle tip to the center of the first dwell of 8.1 mm (4.6 mm from the solid needle tip and 3.5 mm from the inner wall of the needle tip to the center of the first dwell) due both to the difficulty visualizing the extremely pointed tip of the needle and to the MR slice thickness.

Another issue is potential fish wire pull back from the end of the needle, which may be seen if the fish wire was not fully inserted or shifted before the CT scan. This pull back can often be seen in the CT and potentially confirmed on a high quality MR by measuring the MR signal void past the end of the needle. If the length of the MR signal void is greater than the amount shown in Figure 8c, it indicates that the fish wire may have pulled back from the end of the needle. This may be enough to correct for the pull back during digitization, but an additional safety measure may include choosing a CT scan range that includes the hubs of the needles so that if there is any question about the location of the tip of a needle, the entire length of the needle may be digitized to eliminate the confusion. Even careful taping of the radio opaque marker at the open end of the needle does not always prevent this, so optimal treatment planning would utilize a CT/MR registration. Any clinic desiring to use a single imaging modality would have to use an extended CT range or fail‐safe method for securing radio opaque markers for CT‐only planning, or would have to establish a procedure using MR markers for MR‐only techniques that addresses the difficulty and uncertainly in determining needle tips.

An additional potential issue is rotation of the applicator when performing CT/MR registration. If not all needle guide tracks are used or if the needles are inserted to differing enough extents, those asymmetries provide a benchmark to make sure the image registration is performed correctly. However, if the needles are all used and are either not extended into tissue or are all inserted into tissue approximately the same amount, it may be beneficial to use an MR marker to ensure total confidence that the image registration is orientated correctly.

For inventory and tracking purposes, all UIC shells and cores are kept together as sets and tracked through serial numbers. The UCP applicator sets are also inventoried through their respective serial numbers. With the plastic interstitial needles, however, although the serial numbers of all needles were noted and tracked for sterilization records as these needles are cleared for only 25 sterilization cycles, specific commissioning results were not associated with specific serial numbers. Needles were commissioned and released for clinical use in batches with enough frequency to replace needles taken out of service when they reached their sterilization cycle limit.

## 5 CONCLUSION

It is critical to fully commission applicators before their clinical use. We performed a comprehensive set of tests on all components of the UIC applicator set and then additional tests on one combination of components in an attempt to simulate the conditions of their eventual clinical use. The UIC applicator system was commissioned for CT and MR image based treatment planning for HDR brachytherapy. Planar MV and kV images as well as SA models and CT scans verified dimensions of the applicator components against vendor specifications and confirmed the coincidence of SA and physical source channels. Accurate source position reconstruction within the needles was feasible using a CT scan with 0.6 mm slice thickness and was best visualized with the use of nylon coated stainless steel leader wires (“fish wire”) placed in each needle. An MR marker can be used in cases where additional information is required when registering CT and MRI images, such as when all needle channels are used symmetrically and additional confirmation is desired that the applicator has not been rotated. Compared to a liquid or gelatin based phantom, a ground beef phantom minimized unintended MR and CT signal similar to a clinical case by limiting the introduction of fluid into the needle guide tracks of the UIC.

## AUTHOR CONTRIBUTIONS

The authors confirm contribution to the paper as follows: Conception and design of the paper: Sheridan Meltsner, Yongbok Kim, and Oana Craciunescu. Interpretation of results and data: Sheridan Meltsner, Yongbok Kim, Oana Craciunescu, Julie Raffi, Yang Sheng, Casey Lee, Diandra Ayala‐Peacock, and Junzo Chino. Draft manuscript preparation: Sheridan Meltsner. All authors reviewed the results and approved the final version of the manuscript.

## CONFLICT OF INTEREST STATEMENT

The authors declare no conflicts of interest.
